# Influence of SPK with Enteric Drainage on the Pancreatic Exocrine Function in Diabetic Patients with Uremia

**DOI:** 10.1155/2017/3709306

**Published:** 2017-08-24

**Authors:** Guanghui Pei, Wu Lv, Xiaohang Li, Guoqing Zhang, Jialin Zhang

**Affiliations:** ^1^Department of Hepatobiliary and Transplantation Surgery, The First Affiliated Hospital of China Medical University, Shenyang, Liaoning Province, China; ^2^Department of Organ Transplantation, Tianjin First Central Hospital, Tianjin, China; ^3^Department of General Surgery, Liaoning Cancer Hospital and Institute, Shenyang, China

## Abstract

**Objective:**

This study aimed to determine the use of fecal elastase in evaluating the effect of simultaneous pancreas–kidney transplantation with enteric drainage on the pancreatic exocrine function of diabetic patients with uremia.

**Methods:**

A total of 19 patients with simultaneous pancreas–kidney transplantation (SPK) with enteric drainage, 31 diabetic patients with uremia (chronic renal failure (CRF)), 22 diabetic patients with uremia who underwent renal transplantation (RT), and 20 normal individuals (CON) were included in the study. Pancreatic exocrine insufficiency was determined using fecal elastase. Results. The fecal pancreatic elastase level in SPK patients with enteric drainage was 479 *μ*g/g, which was significantly higher than 229 *μ*g/g in CRF patients and 197 *μ*g/g in RT patients. Using 200 *μ*g/g as the established threshold, a reduced fecal pancreatic elastase level was found in 14/31 of CRF patients, 12/22 of RT patients, 1/19 of SPK patients with enteric drainage, and 1/20 of CON patients. The correlation analysis revealed a significant association between fecal elastase and glycosylated hemoglobin.

**Conclusions:**

The present study indicated that SPK with enteric drainage improves pancreatic endocrine and exocrine functions. Fecal elastase may be a clinically relevant means to determine the therapeutic effects.

## 1. Introduction

The literature reports that 20%–80% of diabetic patients and more than 60% of patients with chronic renal failure have an impaired pancreatic exocrine function and varying histological changes in the pancreas [1–10]. The pancreatic exocrine function can be evaluated in two common ways: direct and indirect. Although direct methods employing pancreozymin and secretin have already been the gold standard, they are technically challenging, difficult to standardize, more time consuming, and discomforting to patients following simultaneous pancreas–kidney transplantation.

Elastase is an acidic protease present in the pancreatic juice and feces. It is not degraded in the human intestine. Therefore, it is typically five to six times more concentrated in the feces than in the pancreatic juice. Fecal elastase has been closely correlated with the pancreatic juice elastase and hence reflects the pancreatic exocrine activity. Further, the results of the test are not affected by the pancreatic enzyme replacement therapy. Therefore, measuring fecal elastase can be used as a novel, noninvasive, low-cost, sensitive, and specific method to indirectly determine the pancreatic exocrine function [7, 11–13].

Simultaneous pancreas–kidney transplantation is an effective strategy for treating diabetic patients with uremia. Following the procedure, the pancreatic juice can be drained via two routes: enteric and bladder, with the former being preferred at present. Studies have shown that simultaneous pancreas–kidney transplantation with enteric drainage could effectively improve the pancreatic endocrine function and reduce the complications of diabetes mellitus [14–17]. However, little is known regarding its impact on the exocrine function of the pancreas. In this study, fecal elastase was chosen as a means to evaluate whether simultaneous pancreas–kidney transplantation with enteric drainage could improve the pancreatic exocrine function in diabetic patients with uremia.

## 2. Materials and Methods

### 2.1. Clinical Data

#### 2.1.1. Basic Information

Patients with the following conditions were excluded from this study: pancreatitis, upper abdominal discomfort with unknown causes, gastrectomy, partial small bowel resection, chronic inflammatory bowel disease, any colon disorders, functional dyspepsia, insulinoma, gastrinoma, and/or biliary tract disease. All the patients had normal and stable pancreas and renal function for more than 6 months after simultaneous pancreas–kidney transplantation or kidney transplantation.

All patients were followed up in the outpatient center of Tianjin First Central Hospital. Organs were obtained from donors after cardiac death (DCD) or donors after brain plus cardiac death (DBCD). Informed consents were obtained from all patients. This study was approved by the ethics committee of Tianjin First Central Hospital (number 2016N054KY).

#### 2.1.2. Patient Grouping and Comparison

The present study included 19 patients (16 males and 3 females) with simultaneous pancreas–kidney transplantation (SPK) with enteric drainage, involving 11 patients with type 1 diabetes mellitus (T1DM) and 8 patients with type 2 diabetes mellitus (T2DM) both with uremia; 31 diabetic patients (21 males and 10 females) with uremia (chronic renal failure (CRF)), involving 7 patients with T1DM and 24 patients with T2DM; 22 diabetic patients with uremia (13 males and 9 females) who underwent renal transplantation (RT), involving 3 patients with T1DM and 19 patients with T2DM; and 20 (13 males and 7 females) normal individuals (control (CON)) (Table 1). Demographic parameters, medical history, and laboratory data were collected from all patients, including age, sex, body mass index (BMI), blood glucose, serum insulin, plasma C-peptide, glycosylated hemoglobin, and years of inflicting diabetes mellitus.

The demographic factors were analyzed, and patient's gender, BMI, and etiology of renal failure were found to have no statistically significant difference among the four groups.

#### 2.1.3. Reagents and Materials

The following reagents and materials were used in the study: pancreatic elastase (Bio-Serv, Rostock, Germany), buffers, microplate reader (Thermo Multiskan MK3, USA), electronic scale (Changzhou, China), and deionized water.

## 3. Methods

### 3.1. Fecal Elastase Test

Stool specimens (5 g) were collected in the morning, stored at −80°C, and thawed to room temperature prior to testing. Then, 50 mg of stool specimen was transferred to a 12 mL vial and dissolved in 5 mL of diluted extraction buffer. The suspension was mixed by oscillation and stored at 2–8°C overnight. The fecal elastase level was detected by the enzyme-linked immunosorbent assay (Bio-Serv, Rostock, Germany).

### 3.2. Diagnostic Criteria of Pancreatic Exocrine Insufficiency

Pancreatic exocrine insufficiency was diagnosed according to the manufacturer's instructions (Bio-Serv, Rostock) as follows: >200 *μ*g/g in the immunoassay was considered as normal, 100–200 *μ*g/g was considered as moderately insufficient, and <100 *μ*g/g was deemed severely insufficient in pancreatic exocrine function.

### 3.3. Statistical Analyses

Categorical variables of clinical characteristics of study groups were expressed as frequencies (percentages). The comparisons of the constituent ratio of groups were analyzed using the chi-square test or the Cochran-Mantel-Haensel (CMH) test, as appropriate. Continuous variables were expressed as medians (interquartile ranges). The Mann–Whitney *U* test was used for the comparisons between two groups, and the Kruskal-Wallis test for the comparisons among multiple groups. The correlation analysis of fecal pancreatic elastase and variables was performed using the Spearman method when the distribution of numerical values was not normal. All analyses were conducted using SPSS 19 (SPSS, IL, USA).

## 4. Results

**Figure 1:**
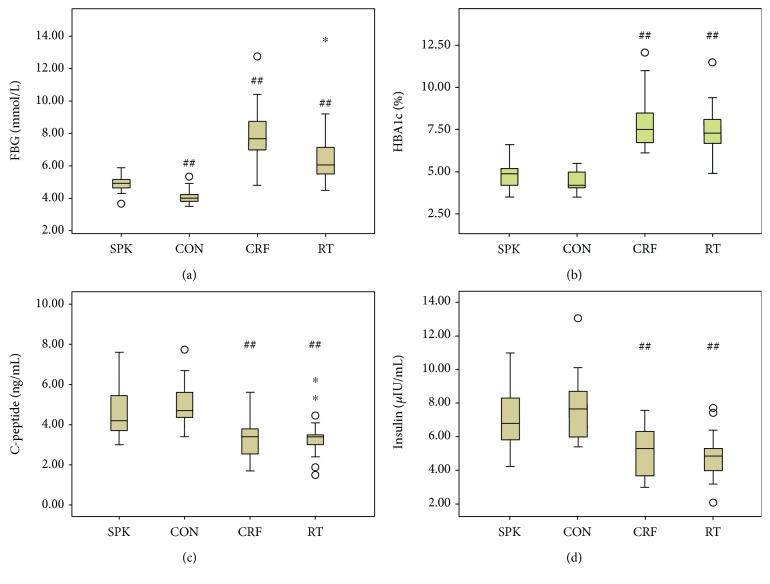
(a–d) Comparison of blood glucose and pancreatic functions among the study groups. ^##^Compared with the SPK group, *P* < 0.01. ∗ presents extreme abnormal value and ○ presents abnormal value.

First, the demographic factors were analyzed, and patient's gender, BMI, and etiology of renal failure were found to have no statistically significant difference among the four groups (the exact *P* values are given in Table 1). The levels of plasma C-peptide, insulin, and glycosylated hemoglobin did not differ in patients receiving simultaneous pancreas–kidney transplantation with enteric drainage (SPK) compared with the normal individuals (CON). Plasma glucose levels of the two groups (CON and SPK) remained within the normal range, although the comparison showed a statistical difference. In contrast, plasma glucose levels in diabetic patients with uremia (CRF) and diabetic patients with uremia who underwent RT both exceeded the normal range, and the CRF group showed a statistically higher abnormal concentration. Next, the duration of diabetes was compared among the last three groups (SPK, CRF, and RT), demonstrating a statistical difference (*P* < 0.001), with the longest duration in the SPK group but no statistically significant difference between CRF and RT (*P* = 0.409). Further, statistically significant differences were observed in the levels of insulin, glycosylated hemoglobin, plasma C-peptide, and blood glucose among SPK, CRF, and RT groups (the exact *P* values are given in Table 2 and Figures 1(a), 1(b), 1(c), and 1(d)).

In the CON group, fecal pancreatic elastase was 441 *μ*g/g. In comparison, elastase was 229 *μ*g/g in CRF patients, 197 *μ*g/g in RT patients, and 479 *μ*g/g in SPK patients. Hence, fecal pancreatic elastase in SPK patients could not be distinguished from that in the CON individuals, but was significantly higher than that in the CRF and RT groups (*P* = 0.003 and *P* = 0.034). As speculated, fecal pancreatic elastase in the RT and CRF groups was lower than that in the CON group, but with no significant difference (*P* = 0.396).

Next, using the 200 *μ*g/g threshold, a reduced fecal pancreatic elastase level was identified in 14/31 of CRF patients, 12/22 of RT patients, 1/19 of SPK patients, and 1/20 of CON patients. Thus, the proportion of lowered elastase was significantly less in SPK patients with enteric drainage than in RT or CRF patients (*P* = 0.006 and *P* = 0.01). Further, fecal pancreatic elastase was consistently reduced in both RT and CRF patients compared with CON patients (*P* = 0.033 and *P* = 0.002).

**Figure 2:**
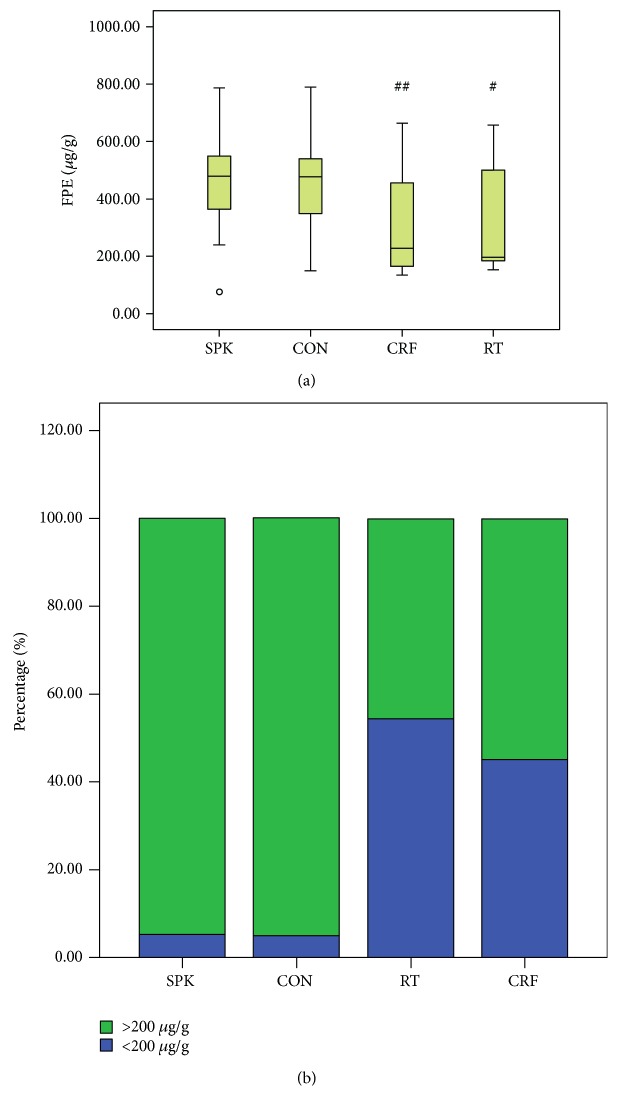
Comparison of the fecal pancreatic elastase level among the study groups. ^#^Compared with SPK group, *P* < 0.05; ^##^compared with SPK group, *P* < 0.01.

Finally, the correlation analysis revealed a negative association between fecal elastase with glycosylated hemoglobin and plasma glucose, a positive association with insulin, and no relationship with BMI, duration of diabetic disease, or plasma C-peptide. SPK patients differed in renal cold ischemic time, immunosuppressive regimen, and induction regimen compared with the RT group. Neither of the two groups had significant differences in lymph toxicity or human leucocyte antigen (HLA) mismatching (Tables 2–4 and Figures 1(a), 1(b), 1(c), and 1(d) and 2(a) and 2(b)).

## 5. Discussion

Patients with diabetes mellitus or uremia are frequently reported to have pancreatic exocrine insufficiency. Estimates show about 56.7% patients with T1DM and 35% patients with T2DM combining with pancreatic exocrine insufficiency [5]. Pancreatic exocrine activity is an important measure of intact pancreatic function. Balo and Banga [11] first described the human pancreatic elastase as an acidic protease that exists in the pancreatic secretions and feces as an endopeptidase and sterol-binding protein. Elastase, along with other pancreatic enzymes, is synthesized by acinar cells. Therefore, reduction in elastase in pancreatic secretions and feces is a sensitive indicator of pancreatic exocrine insufficiency. One study using the correlation analysis indicated that elastase had a significant correlation with the levels of lipase, amylase, trypsin, and bicarbonate in the duodenal juice [13]. Further, detection of fecal elastase was not affected by the urine volume of patients with diabetes and/or uremia and the dialysis frequently needed by these patients. Therefore, fecal elastase represents a better approach to quantify pancreatic exocrine function in these patients. As a matter of fact, the use of enzyme-linked immunosorbent assays to detect fecal elastase has been widely accepted as a benchmark to evaluate pancreatic exocrine insufficiency. Loser et al. [13] studied the stimulatory effects of bombesin in patients with varied pancreatic exocrine insufficiency. They found that using 200 *μ*g/g as the established threshold, the fecal pancreatic elastase had a 93% sensitivity and specificity in detecting pancreatic exocrine insufficiency. The minimal level of fecal elastase detected by this method was 15 *μ*g/g. In another study, Soldan et al. compared 23 normal individuals with 16 patients with cystic fibrosis, wherein the pancreatic exocrine insufficiency was verified using the secretin test. The results indicated that, with a threshold set to 200 *μ*g/g, fecal elastase had 100% sensitivity and 96% specificity in detecting pancreatic exocrine insufficiency [18].

**Figure 3:**
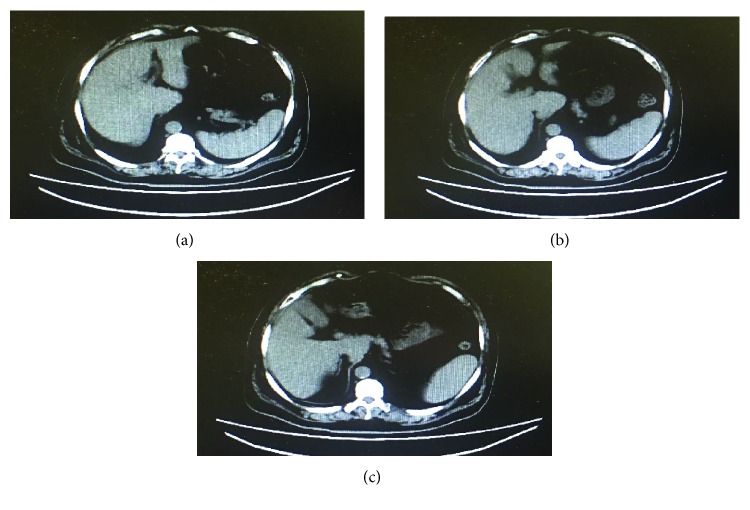
(a–c) Abdominal CT of one patient with the low fecal elastase level (less than 100 *μ*g/g) showed atrophy of the pancreas.

**Figure 4:**
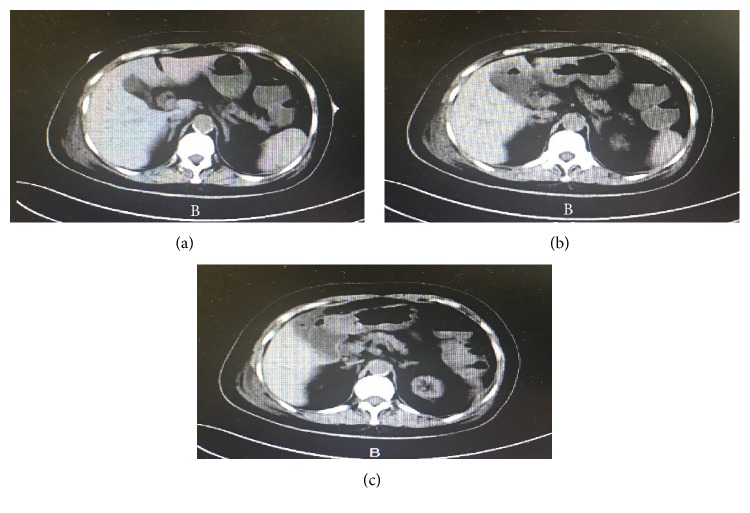
(a–c) Abdominal CT of another patient with the low fecal elastase level (less than 100 *μ*g/g) showed atrophy of the pancreas.

This study used fecal elastase to evaluate pancreatic exocrine function, and the pancreatic exocrine insufficiency in diabetic patients with uremia was confirmed. Multiple factors have been implicated in the process. First, insulin deficiency may cause pancreatic exocrine insufficiency in diabetic patients. Insulin has been considered by some scholars as a nutrient to pancreatic acinar cells. Islet cells supply blood and oxygen to nearby acinar cells. As a result, acinar cells adjacent to the vasculature are greater in cellular volume and more active in secreting digestive enzymes. In this sense, insulin not only is a growth factor but also promotes acinar secretion of enzymes [19–21]. However, Larger et al. [22] described that exocrine failure in patients with T2DM was correlated with a higher possibility of being treated with insulin, indicating that insulin has an important negative impact on the pancreatic exocrine function. Second, changes in the gastrointestinal hormones may promote pancreatic exocrine insufficiency. Reports have shown that high levels of glucagon and somatostatin in diabetic patients could reduce pancreatic exocrine function [23, 24]. Third, autoimmunity in diabetic patients may also induce pancreatic exocrine insufficiency [25]. Many studies have shown significant morphological changes in the exocrine pancreas of patients with diabetes mellitus. The changes are more notable in patients with T1DM than in patients with T2DM because these individuals have significantly smaller and more atrophic pancreas [26]. Moreover, in several patients with low fecal elastase levels, abdominal computed tomography (CT) showed atrophy of the pancreas (Figures 3 and 4). Finally, other concurrent diseases might also affect pancreatic exocrine function, including pancreatic cancer, cystic fibrosis, and diabetic neuropathy [27, 28].

It has long been appreciated that uremic patients frequently have pancreatic exocrine insufficiency. In fact, two-thirds of patients with chronic renal failure were reported to have varied impairment in pancreatic exocrine competency and histological changes in the pancreas. The present study showed that the level of fecal pancreatic elastase was significantly higher in patients with simultaneous pancreas–kidney transplantation than in patients who did not undergo the procedure and higher than in patients with kidney transplantation. This confirmed the hypothesis that simultaneous pancreas–kidney transplantation could not only significantly improve the pancreatic endocrine function but also restore exocrine function in these patients. These findings indicated that simultaneous pancreas–kidney transplantation was especially suitable for diabetic patients with uremia having severe pancreatic exocrine dysfunction. This could be explained on the basis of the enzymes secreted by the transplanted pancreas. Further, enteric drainage allows the secretions of transplanted pancreas to flow directly into the jejunum and mix with jejunal contents, thereby executing pancreatic exocrine function.

The present study showed that fecal elastase level in patients with simultaneous pancreas–kidney transplantation with enteric drainage was significantly higher and not statistically different from that in the healthy population, demonstrating that the procedure could improve the pancreatic exocrine insufficiency in these patients. In contrast, the fecal elastase level in diabetic patients with uremia who underwent kidney transplantation alone was not statistically different from that in the patients who did not undergo the procedure. This indicated that the increase in fecal elastase was caused by the transplanted pancreas rather than the kidney. The correlation analysis revealed no association between pancreatic exocrine function and diabetic course, BMI, or C-peptide, but an association with the glycosylated hemoglobin. However, still some controversies exist. In the present study, a negative association was identified between fecal elastase and glycosylated hemoglobin. This was similar to some studies that showed a negative correlation between fecal elastase and HbA1c, but few others failed to show any association [29, 30]. Also, conflicting reports are available on the association between pancreatic exocrine function and BMI. The present study showed no association between pancreatic exocrine function and BMI. However, the study by Shivaprasad et al. showed a positive correlation between pancreatic exocrine function and BMI [22, 30]. The difference between the findings of this and other studies may be caused by different ethnicity of the patients.

This study still had some limitations. Being a retrospective study, it did not detect the elastase level before SPK transplantation. Moreover, the modality of transplantation was chosen mainly depending on the economic condition, wishes, and surgical tolerance of the patients. Perhaps, this would have accounted for some differences among the patients in these groups. Large-sample studies are needed to validate the findings.

Collectively, this study confirmed that diabetic patients with uremia also had pancreatic exocrine dysfunction. The findings suggested that simultaneous pancreas–kidney transplantation with enteric drainage could restore the pancreatic exocrine insufficiency. In comparison, RT alone might not be sufficient and fecal elastase might be a clinically relevant means to determine the therapeutic effects. Glycosylated hemoglobin may be associated with pancreatic exocrine insufficiency. Future studies need to address whether simultaneous pancreas–kidney transplantation with enteric drainage could affect the levels of amino acids, fat-soluble vitamins, and gastrointestinal hormones.

## Figures and Tables

**Table 1:** Clinical characteristics of study groups.

Variable	SPK (*n* = 19)	RT (*n* = 22)	CRF (*n* = 31)	CON (*n* = 20)	*P* ^∗^
Gender, *n* (%)					0.362
Male	16 (84.2)	13 (59.1)	21 (67.7)	13 (65.0)	
Female	3 (15.8)	9 (40.9)	10 (32.3)	7 (35.0)	
Type of diabetes mellitus, *n* (%)					0.004
T1DM	11 (57.9)	3 (13.5)	7 (22.6)	—	
T2DM	8 (42.1)	19 (86.5)	24 (77.4)	—	
Etiology of renal failure (%)					0.172
Chronic glomerulonephritis	6 (31.6)	15 (68.2)	21 (67.7)	—	
Diabetic nephropathy	7 (36.8)	3 (13.6)	6 (19.4)	—	
Hypertensive nephropathy	3 (15.8)	3 (13.6)	3 (9.7)	—	
Others	3 (15.8)	1 (4.5)	1 (3.2)	—	
Age (year), median (IQR)	48 (40–54)	49 (46–54)	51 (45–58)	42 (33–48)^#^	0.003
BMI (kg/m^2^), median (IQR)	24.6 (23.5–26.4)	22.3 (20.4–28.7)	23.4 (21.8–26.2)	24.0 (22.4–25.5)	0.642
Course of DM (year), median (IQR)	20 (18–22)	15 (12–17)^#^	14 (11–15)^#^	—	<0.001

^∗^Comparison among four or three groups; ^#^Compared with the SPK group. SPK: simultaneous pancreas–kidney transplantation; CRF: diabetic patients with uremia (chronic renal failure); RT: diabetic patients with uremia who underwent renal transplantation; CON: normal individuals; T1DM: type 1 diabetic mellitus; T2DM: type 2 diabetic mellitus; BMI: body mass index; DM: diabetes mellitus.

**Table 2:** Comparison of blood glucose and pancreatic islet function among study groups.

Variable	SPK (*n* = 19)	RT (*n* = 22)	CRF (*n* = 31)	CON (*n* = 20)	*P* ^∗^
Blood glucose (mmol/L), median (IQR)	5.0 (5.6-5.2)	6.1 (5.4–7.3)^##^	7.7 (6.9, 8.8)^##^	4.0 (3.8-4.3)^##^	<0.001
Glycosylated hemoglobin (%), median (IQR)	4.8 (4.2-5.2)	7.3 (6.7–8.3)^##^	7.5 (6.7–8.8)^##^	4.2 (4.0-5.0)	<0.001
Insulin (*μ*IU/mL), median (IQR)	6.8 (5.8–8.3)	4.8 (4.0-5.3)^##^	5.3 (3.6–6.4)^##^	7.7 (6.0–8.8)	<0.001
C-peptide (ng/mL), median (IQR)	4.2 (3.6–6.5)	3.4 (2.9-3.5)^##^	3.4 (2.5–4.0)^##^	4.7 (4.3-5.7)	<0.001
FPE (*μ*g/g), median (IQR)	479 (335–557)	197 (185–502)^#^	229 (163–457)^##^	441 (341–554)	0.003
<200 *μ*g/g (%)	1 (5.3)	12 (54.5)	14 (45.2)	1 (5.0)	<0.001
≧200 *μ*g/g (%)	18 (94.7)	20 (45.5)	17 (54.8)	19 (95.0)	

^∗^Comparison among four groups; ^#^compared with the SPK group, *P* < 0.05; ^##^compared with the SPK group, *P* < 0.01. SPK: simultaneous pancreas–kidney transplantation; CRF: diabetic patients with uremia (chronic renal failure); RT: diabetic patients with uremia who underwent renal transplantation; CON: normal individuals; FPE: fecal pancreatic elastase.

**Table 3:** Clinical information of SPK and RT transplantation groups.

Transplantation	SPK (*n* = 19)	RT (*n* = 22)	*P*
Pancreatic cold ischemic time (h), median (IQR)	6 (6-7)	—	—
Renal cold ischemia time (h), median (IQR)	4 (3–5)	8 (5–9)	0.000
HLA mismatch, median (IQR)	2 (2-3)	3.0 (2.0-3.3)	0.693
Negative lymphocytotoxicity, *n* (%)	19 (100)	22 (100)	—
Immunosuppressive regimen, *n* (%)			<0.001
FK506 + MMF + Pred	17 (89.5)	3 (13.6)	
CsA + MMF + Pred	2 (10.5)	15 (68.2)	
RAPA + MMF + Pred	0 (0.0)	4 (18.2)	
Induction regimen, *n* (%)			<0.001
Simulect	2 (10.5)	20 (90.9)	
ATG	17 (89.5)	2 (9.1)	

SPK: simultaneous pancreas–kidney transplantation; RT: diabetic patients with uremia who underwent renal transplantation; HLA: human leucocyte antigen; FK506: tacrolimus; MMF: mycophenolate mofetil; Pred: prednisone; CsA: ciclosporin; RAPA: rapamycin; ATG: rabbit anti-human thymocyte globulin.

**Table 4:** Correlation analysis of fecal pancreatic elastase and variables.

Variable	FPE	
	Spearman's *r*	*P*
HBA1c	−0.377	<0.001
FBG	−0.320	0.002
C-peptide	0.174	0.097
Length of disease	0.192	0.106
Age	−0.184	0.078
BMI	0.076	0.471
Insulin	0.445	<0.001

FBG: fasting blood glucose; FPE: fecal pancreatic elastase; BMI: body mass index.
